# Clinical risk factors for pancreatic cancer: protocol for an umbrella review

**DOI:** 10.1136/bmjopen-2024-089008

**Published:** 2024-11-07

**Authors:** Sarah Moore, Sarah Price, Gianni Dongo, Fiona M Walter, Richard Neal, Gary A Abel

**Affiliations:** 1 Department of Health and Community Sciences, University of Exeter, Exeter, UK; 2 Wolfson Institute of Population Health, Queen Mary University of London, London, UK

**Keywords:** Risk Factors, Epidemiology, Systematic Review

## Abstract

**Abstract:**

**Introduction:**

Identifying cancer earlier can help save lives. An increasingly popular approach to diagnosing cancer earlier is in the development of risk prediction models to be applied to the electronic healthcare record of patients. Development of these models requires systematic and thorough identification of the risk factors that might increase an individual’s propensity to develop the disease. This protocol sets out the methods for an umbrella review to identify risk factors that might be included in these models. The example used is pancreatic cancer, a disease with a high percentage of late-stage diagnoses and consequent high mortality.

**Methods and analysis:**

Relevant systematic reviews will be identified through searching of MEDLINE and EMBASE via Ovid and the Science Citation Index Expanded of the Web of Science Core Collection. Screening will be performed by two independent reviewers using Covidence software and the results reported as a Preferred Reporting Items for Systematic Reviews and Meta-Analyses flow diagram. Data from eligible studies will be extracted independently by two reviewers and each systematic review will be graded using defined credibility assessment criteria and the ROBIS (Risk of Bias in Systematic Reviews) tool. Results will be presented in detail for each paper. Summary results for each risk factor will be discussed in the narrative and summarised using a table, graphical summary and an infographic.

**Ethics and dissemination:**

Ethical approval is not required for this review. Results of the review will be disseminated by publication in a peer-reviewed journal and presented at conferences.

**PROSPERO registration number:**

CRD42024526338.

**Strengths and limitations of this study**

- Using umbrella review methodology will provide a comprehensive overview of the systematic reviews and meta-analyses on individual clinical risk factors for pancreatic cancer.
- Thorough assessment of strength of evidence and quality of included reviews will increase the robustness of the results.
- There will be some overlap of studies used by systematic reviews on the same topic. This will be mitigated by using strength of evidence assessments to decide which studies to include in the final results summary.

## Introduction

 Pancreatic cancer is increasingly common and its survival universally poor, for example, in the UK it is the 10th most common cancer and has a survival rate of only 5% at 5 years.^1^ This is in part due to most cases being diagnosed at a late stage, when the cancer has spread beyond the pancreas and the prognosis is worse.^2^

One hope for improving pancreatic cancer survival is therefore to identify it at an earlier stage, when it has not spread and is more treatable. An increasingly popular way of doing this is through using electronic healthcare records to develop models that identify people at current or future high risk of pancreatic cancer.^3^ Identification of either group can help improve early diagnosis, though the mechanisms for doing so are different. The first, identifying a high risk of undiagnosed current cancer, allows for earlier investigation and potential subsequent earlier diagnosis. The second, identifying people with a high future risk of cancer, means it is possible to initiate screening and/or surveillance as well as implement preventative action. In order to develop either of these model types, it is vital to have an understanding of the key risk factors for pancreatic cancer, as these will form the pool of candidate variables for model development (see box 1 for disambiguation of terms factor, variable and feature).

Box 1 **Defining terms: features, factors and variables**

In the literature surrounding risk prediction models, several terms are used interchangeably and can cause confusion. We will therefore clarify how we are using each term for the purposes of this paper.

A dataset is made up of information about an individual, for example, their age, what medications they take or whether they are smokers. Each piece of information is known as a *variable* and some of these will be of potential relevance to the model and others will not. Those that are identified as potentially relevant are known as the *candidate variables*, from which the *final variables* that will form the basis for the model will be chosen using statistical or machine learning techniques.

*Risk factors*, sometimes referred to simply as *factors*, are variables that are associated with cancer development. A classic example is smoking. Risk factors affect the prior odds of an individual developing cancer. In this review, we are identifying *risk factors* for pancreatic cancer that can be used as *candidate variables* for a risk prediction model.

*Features* refers to the signs, symptoms or test results that could indicate an undiagnosed cancer is present. These are not being investigated in this paper but will be important for development of models of current undiagnosed cancer risk for symptomatic patients. NB: The term features is often used in machine leaning literature to refer to variables.

A systematic review of pancreatic cancer prediction models identified 33 articles describing 38 models predicting the risk of pancreatic cancer.^3^ Although they summarised which factors were ultimately included in each model, further exploration of the studies behind the models shows no consistent approach to identifying the candidate variables from which the model can be built.^3^ There are thousands of potential risk factors available in electronic healthcare records and these need to be refined when developing a model, in order to achieve the most accurate prediction.^4^ There is therefore a significant need for developing a robust process for identifying potential candidate variables from which the final features can be selected. This is usually performed based on subject knowledge and, in some cases, systematic review, followed by statistical or machine learning-led selection to define the final variables for inclusion in the model.^5^

The candidate variables that can practically be used in these models at present (certainly in the UK setting) are those available to researchers using large databases of coded electronic healthcare records, though in some places this has already been expanded to include free text information using natural language processing capabilities.^6^ Although research datasets such as UK Biobank may contain information on genetics or novel biomarkers,^7^ the records used in routine clinical care at present do not. In addition to this, there is limited access in routine healthcare data to information on diet and these factors have already been explored in recent comprehensive reviews.^8 9^

Given the wealth of literature available on potential risk factors for pancreatic cancer, this study will take the approach of an umbrella review, which systematically identifies and assesses multiple systematic reviews and meta-analyses on a specific topic to provide an overall picture.^10 11^

The last comprehensive summary review of meta-analytical studies examining clinical risk factors for pancreatic cancer was published nearly 10 years ago and since then there have been a significant number of new systematic reviews looking at individual risk factors for pancreatic cancer.^12^ In addition, the previous review of reviews used a very simplified format for grading the strength of evidence for each association compared with the criteria for credibility assessments used in many umbrella-type reviews.^12 13^ It is, therefore, timely to repeat and expand this review of reviews, considering the needs of researchers using the findings for the development of risk prediction models using electronic healthcare records.

### Objectives

The primary objective of this umbrella review is to identify potential risk factors for pancreatic cancer in adults which are accessible to clinicians and healthcare researchers in the electronic healthcare record. Secondary objectives comprise quantification of the magnitude of the effect and a description of the strength of the evidence for each risk factor.

## Methods

### Design and registration

This protocol has been developed in accordance with the Preferred Reporting Items for Systematic Review and Meta-Analysis Protocols^14 15^ (see online supplemental appendix 1). Guidance from the Joanna Briggs Institute, Cochrane collaboration and other published sources on the methodology underpinning systematic and umbrella reviews have been taken into account in its development.^10 11 16 17^ It has been registered with PROSPERO (registration number: CRD42024526338).

### Eligibility criteria

These are based on the PECOS statement^18^ (see table 1).

**Table 1:** Eligibility criteria for studies to be included in the umbrella review

Population	Adults over the age of 18 years
Exposure	Any factors which might influence the development of pancreatic cancer and are available in an electronic healthcare record
Comparator	Adults who have not been exposed, or have been differentially exposed, to the factors which might influence the development of pancreatic cancer
Outcome	Diagnosis of pancreatic cancer
Study designs	Systematic reviews and meta-analyses
Setting	Any setting

Reviews will be eligible for inclusion if they are systematic reviews or meta-analyses of component studies with suitable epidemiological design, for example, cohort or case–control studies, they will not be eligible if theoretical studies or published opinion are their primary source of evidence. Eligible reviews will examine risk factors for pancreatic cancer that could be available in a coded electronic patient healthcare record and will therefore exclude factors that require genome sequencing or use of novel biomarkers. The cancers of interest are primary cancers of the pancreas in adults. Studies solely examining neuroendocrine tumours will be excluded. There will be no geographical or time restriction on the included reviews, but they will be excluded if there is no full text of the completed study available in the English language.

### Information sources

Systematic searches will be performed on MEDLINE and EMBASE via Ovid and the Science Citation Index Expanded database on the Web of Science Core Collection. Supplementary searches including forward and backward citation chasing will be performed through Scopus. The Cochrane database has not been included as their focus is on interventional rather than observational studies. Grey literature is not being searched as it is very unlikely to be a source of systematic reviews.

### Search strategy

Key search concepts are ‘pancreatic cancer’, ‘risk factors’ and ‘systematic reviews’. Full details of the exact search terms to be used can be found in online supplemental appendix 2.

### Study records

Covidence software for managing systematic reviews (https://www.covidence.org/) will be used for screening abstracts and full texts. Two independent reviewers will screen all records retrieved for eligibility. Data from eligible studies will be extracted into preformatted tables by two independent reviewers and compared. Throughout, any disagreement between the two reviewers will be identified and resolved by discussion until consensus is reached, and if this is not possible a third reviewer will be consulted. In circumstances where required data are not available then the authors of the original review will be contacted for clarification. If, after a second approach, this is not possible then the review will be included but marked as having missing information.

### Data items

Data will be extracted under multiple headings, as shown in table 2.

**Table 2:** Data extraction fields for each eligible review

Column heading	Explanation
Risk factor	NB: If a review explores more than one risk factor it will be included multiple times in the extraction, once for each factor.
Reference	To include lead author, year, DOI
Type of review	For example, pooled/meta-analysis
Number/type of studies	For example, cohort/case–control
Number of cases/controls	Across the whole review/meta-analysis
Population characteristics	For example, location, sex
Strata	Difference between exposed and comparator populations, for example, 5-unit increase in BMI or BMI above or below 30
Effect size metric	For example, relative risk/OR/HR
Summary effect size (95% CI)	As per the study findings
P value for summary effect estimate	As per the study findings
Measure of heterogeneity	For example, I^2^, as per the study findings
Grading of strength of evidence	Based on criteria for credibility of assessment (see below)
Notes	Any extra notes from the extractor on the review

BMIbody mass index

Most data will be extracted directly from the identified reviews but grading the strength of evidence and quality of the reviews will be completed separately as part of the process.

#### Grading strength of evidence for each association

There is no consensus on the best method for grading strength of evidence in an umbrella review. In a scoping review of what has been used previously, only half of studies assessed certainty of the evidence, and within those studies the most commonly used criterion was credibility assessment (80%), followed by the GRADE (Grading of Recommendations Assessment, Development and Evaluation) approach (14%).^13^

Credibility assessment criteria were similar between studies but the levels at which they met a threshold varied slightly depending on the study.^13^ We have used the most commonly occurring criteria and thresholds, as identified in Sadoyu *et al*^13^ and recommended in Fusar-Poli and Radua,^19^ as the basis for our credibility assessment criteria (see table 3).

**Table 3:** Credibility assessment criteria for this study, derived from findings of Sadoyu *et al*^13^

Measure	Threshold			
	Convincing	Highly suggestive	Suggestive	Weak
Number of cases	>1000 cases	>1000 cases	>1000 cases	
P value	<10^−6^	<10^−6^	<10^−3^	<0.05
Heterogeneity	I^2^<50%	–	–	–
Largest study with statistically significant effect	Largest study nominally significant (p<0.05)	–	–	–

Given the primary aim of this review is the identification of *potential* risk factors, we will not be deriving 95% prediction intervals, evidence of small-study effects or evidence of excess significance bias in order to assess publication and other biases within the component studies of a systematic review, nor including them in our credibility assessment criteria.

#### Assessing methodological quality of reviews

For the type of reviews included in this study, the best available method for assessing quality is the ROBIS (Risk of Bias in Systematic Reviews) tool which includes four key domains: study eligibility criteria, identification and selection of studies, data collection and study appraisal and synthesis and findings.^20 21^ This tool was chosen as it has been shown to perform better in the assessment of meta-analyses which we anticipate will form the majority of our included papers.^22^ The ROBIS tool will be completed for each included study and summary shown in the final detailed results table.

### Other data considerations

#### Comparing the effect sizes

Effect size is a measure of the strength of the relationship between the risk factor and the development of the disease. Effect size is the main quantitative outcome of interest for this study and it is important that effect sizes can be compared between risk factors.^19^ Although not all studies use the same measures to report their effect size, we can treat the likely reported measures of relative risk, HRs, ORs and incidence rate ratios as approximately equal because the event rate for pancreatic cancer is typically less than 10%.^19 23^

#### Multiple reviews on the same risk factor

There are likely to be multiple reviews on the same risk factor and there is no consensus on how to deal with overlapping reviews.^16 24^ In the previous review of reviews of the topic in 2015, the authors averaged the risk estimates reported in all available meta-analyses and pooled analyses.^12^ However, this leads to a risk of including multiple component studies more than once, as they occur repeatedly in each review. Given the aim of that study was simply to *identify* potential risk factors, overlap of included studies did not matter. However, there remains the issue of the strength of the evidence in each study and the risk of the results of smaller high-quality analyses being diluted by large poor-quality studies. To avoid this, we will use an alternative common approach to overlapping studies, which is to select the single largest, most recent or highest quality meta-analysis or systematic review to represent the relationship between the exposure and outcome.^24^ Our priority is to identify robust relationships, and we therefore propose that, in the event of multiple reviews of the same risk factor, once data have been extracted for each study, we will select the study with the highest strength of evidence according to our credibility assessment criteria grading (see previous section). If there is more than one review with ‘convincing’ evidence, we will select from them the review with the best quality according to the ROBIS assessment. If this still results in more than one study, we will select that with the largest pooled number of participants.

### Outcomes and prioritisation

The main outcome will be a list of risk factors for pancreatic cancer that can be defined in coded electronic healthcare records. Additional outcomes will be the strength of the effect of the risk factor and the strength of the evidence for the effect, according to the criteria described above.

### Data synthesis

In this umbrella review, quantitative synthesis will not be performed, instead summary results for each risk factor will be presented in a table (see table 4 for key headings) and discussed in the narrative. A graphical summary will be developed from the key results to show direction and magnitude of reported effect sizes and a simple infographic grouping the factors by section, for example, demographic, lifestyle, medical history.

**Table 4:** Summary results table plan (results of the main selected study on each factor)

Column heading	Explanation
Risk factor	Description of factor
Degree of association	Measured by relative risk in largest study of good quality
Strength of evidence	As per credibility assessment criteria above

### Patient and public involvement

The patient and public involvement panel, already recruited to the overarching study, will contribute to discussions around the findings of this umbrella review in a designated session. Their thoughts will be integrated into the final write-up of the study.

### Ethics and dissemination

Ethical approval is not required to perform this review.

Results of the study will be published in a peer-reviewed journal and presented at academic conferences. All collected data will be made available as appendices to the published paper.

### Data statement

All data generated will be available as appendices to the final published study report.

## supplementary material

10.1136/bmjopen-2024-089008 online supplemental appendix 1

10.1136/bmjopen-2024-089008 online supplemental appendix 2
