# Supplementary material for: Clinical risk factors for pancreatic cancer: protocol for an umbrella review
Source: BMJ Open. 2024 Nov 7;14(11):e089008. doi: 10.1136/bmjopen-2024-089008 (PMC11552553; doi:10.1136/bmjopen-2024-089008)
Supplement: online supplemental appendix 1 [file bmjopen-14-11-s001.docx]

**Appendix 2: Specific search terms by database and platform**

**MEDLINE via Ovid**

1 (pancrea* neoplasm* or (pancrea* adj3 carcinoma*) or (Cancer adj3 pancrea*) or (Malignan* adj3 pancrea*) or (Pancrea* adj3 carcinogenesis) or (Pancrea* adj3 tumo?r)).af. or Pancreatic neoplasms.sh.

2 (risk factor* or health correlate* or population* at risk or precipitating factor* or sociodemographic factor* or protective factor* or epidemiologic* factor* or epidemiologic* determinant* or relative risk).ti,ab,mp. or Risk Factors.sh. or Protective factors.sh. or Epidemiologic factors.sh.

3 (systematic review* or umbrella review* or meta?analys* or meta regression or meta analys* or medline or pubmed).ti,ab,mp. or meta-analysis.sh. or systematic review.sh.

4 1 and 2 and 3

**EMBASE via Ovid**

1 (pancrea* neoplasm* or (pancrea* adj3 carcinoma*) or (Cancer adj3 pancrea*) or (Malignan* adj3 pancrea*) or (Pancrea* adj3 carcinogenesis) or (Pancrea* adj3 tumo?r)).af. or Pancreas cancer.ec. or Pancreas carcinoma.ec.

2 (risk factor* or health correlate* or population* at risk or precipitating factor* or sociodemographic factor* or protective factor* or epidemiologic* factor* or epidemiologic* determinant* or relative risk).ti,ab,mp. or risk factor.ec. or protection.ec.

3 (systematic review* or umbrella review* or meta?analys* or meta regression or meta analys* or medline or pubmed). ti,ab,mp. or Meta analysis.ec. or Systematic review.ec.

4 1 and 2 and 3

**Science Citation Index Expanded on Web of Science Core Collection**

1: (((((TS=(pancrea* neoplasm*)) OR TS=(pancrea* NEAR/3 carcinoma* )) OR TS=(Cancer NEAR/3 pancrea* )) OR TS=(Malignan* NEAR/3 pancrea* )) OR TS=(Pancrea* NEAR/3 carcinogenesis )) OR TS=(Pancrea* NEAR/3 tumo$r )

2: ((((((((TS=(risk factor*)) OR TS=(health correlate*)) OR TS=(population* at risk)) OR TS=(Precipitating factor*)) OR TS=(Sociodemographic factor*)) OR TS=(Protective factor*)) OR TS=(Epidemiologic* factor*)) OR TS=(Epidemiologic* determinant*)) OR TS=(Relative risk)

3: ((((((((TS=(systematic review*)) OR TS=(umbrella review*)) OR TS=(Meta-analys*)) OR TS=(Meta regression)) OR TS=(Meta analys*)) OR TS=(Metaanalys*)) OR TS=(Medline*)) OR TS=(Pubmed*))

4: #1 AND #2 AND #3
