# Supplementary material for: Clinical risk factors for pancreatic cancer: protocol for an umbrella review
Source: BMJ Open. 2024 Nov 7;14(11):e089008. doi: 10.1136/bmjopen-2024-089008 (PMC11552553; doi:10.1136/bmjopen-2024-089008)
Supplement: Supplementary file 3 [file bmjopen-2024-089008.reviewer_comments.pdf]

PEER REVIEW HISTORY

BMJ Open publishes all reviews undertaken for accepted manuscripts. Reviewers are asked to complete a checklist review form and are provided with free text boxes to elaborate on their assessment. These free text comments are reproduced below.

ARTICLE DETAILS

Title (Provisional)

Clinical risk factors for pancreatic cancer: protocol for an umbrella review.

Authors

Moore , Sarah; Price, Sarah; Dongo, Gianni; Walter, Fiona M; Neal, Richard; Abel, Gary A

VERSION 1 - REVIEW

**Reviewer**	**1**
**Name**	**Wei, Qiang**
**Affiliation**	**Sichuan Univ, Department of urology**
**Date**	**16-Sep-2024**
**COI**	**None.**

Moore et al report on the protocol of an umbrella review aimed at identifying potential risk factors for pancreatic cancer in adults available to clinicians and healthcare researchers in electronic medical records. Before its official publication, the following issues need to be addressed:

1. Did the author consider including the Cochrane database in the literature search? It is well known that the Cochrane database contains a large number of high-quality meta-analyses, which are regularly updated.

2. Why did the author choose ROBIS tool instead of AMSTAR-2, a more commonly used meta-analysis tool, to evaluate the quality of the included literature?

3. It is suggested that the author supplement the possible problems and inevitable limitations of this study, and provide directions for further research.

4. There are a lot of punctuation and grammar errors in the author's search formula, please correct them.

**Reviewer**	**2**
**Name**	**Xue, Kun**

**Affiliation**	**Fudan University School of Public Health**
**Date**	**19-Sep-2024**
**COI**	**no**

The methodological description of the umbrella meta-analysis in this manuscript is good, but the topic selection needs to be modified to avoid repeated work, because some paper of the similar topic has been published as followed:

Diets, Dietary Patterns, Single Foods and Pancreatic Cancer Risk: An Umbrella Review of Meta-Analyses.Int. J. Environ. Res. Public Health 2022, 19(22), 14787;

Dietary Factors and Pancreatic Cancer Risk: An Umbrella Review of Meta-Analyses of Prospective Observational Studies.Adv Nutr. 2023 May; 14(3): 451–464.

VERSION 1 - AUTHOR RESPONSE

Reviewer 1		
2.1	Did the author consider including the Cochrane database in the literature search? It is well known that the Cochrane database contains a large number of high-quality meta-analyses, which are regularly updated.	Thank you for your suggestion. We did consider using the Cochrane database but on reviewing the potential studies available realised it did not suit our clinical question as the focus of Cochrane reviews is on interventional trials, usually using RCTs. Our review focuses on systematic reviews and meta-analyses of observational studies. We have added a sentence to the Information sources section of the methods to clarify this: 'The Cochrane database has not been included as their focus is on interventional rather than observational studies.'
2.2	Why did the author choose ROBIS tool instead of AMSTAR-2, a more commonly used meta-analysis tool, to evaluate the quality of the included literature?	Thank you for raising this point. This was carefully considered and the decision was taken for 2 key reasons.<br>1. There is evidence that ROBIS is better for assessment of meta-analyses, which we expect to form the majority of included papers: (https://systematicreviewsjournal.biomedcentral.com/artic

		les/10.1186/s13643-021-01819-x).<br>2. ROBIS has been designed from the start to include assessment of reviews of observational studies, whereas AMSTAR has a strong history of being only for reviews of RCTs and even now focuses on interventional methods.<br>We have updated the paper to reflect this with the addition of the sentence ‘This tool was chosen as it has been shown to perform better in the assessment of meta-analyses which we anticipate will form the majority of our included papers (20).’ In the section ‘Assessing methodological quality of reviews’.
2.3	It is suggested that the author supplement the possible problems and inevitable limitations of this study, and provide directions for further research.	Thank you for your suggestion.<br>We have changed the strengths and limitations bullet pointed section on page 3, as discussed above in response to the editor point 1.1.
2.4	There are a lot of punctuation and grammar errors in the author's search formula, please correct them.	Thank you for your suggestion. Although the search is not in plain English, what we have included is the full search terms as directly copied from the relevant search engines. We have done this so our searches are fully reproducible and transparent.<br><br>The resulting format inevitably appears odd but the strange grammar and punctuation are a result of the way the search engines work, e.g. a ‘?’ is put in where there may or may not be an additional letter added. More detail is available here from the Joanna Briggs Institute, whose guidance we followed: https://journals.lww.com/ajnonline/

		Fulltext/2014/05000/Systematic_Reviews___Constructing_a_Search.27.aspx
Reviewer 2		
3.1	The methodological description of the umbrella meta-analysis in this manuscript is good, but the topic selection needs to be modified to avoid repeated work, because some paper of the similar topic has been published as followed:<br>Diets, Dietary Patterns, Single Foods and Pancreatic Cancer Risk: An Umbrella Review of Meta-Analyses.Int. J. Environ. Res. Public Health 2022, 19(22), 14787;<br>Dietary Factors and Pancreatic Cancer Risk: An Umbrella Review of Meta-Analyses of Prospective Observational Studies.Adv Nutr. 2023 May; 14(3): 451–464.	Thank you for your feedback.<br><br>In terms of the suggestions about dietary risk factors and overlap with our study, we really appreciate you raising this question, as we realise we have not clarified this in the original text. Our aim was always to include only those factors that could be found in routine electronic healthcare records and dietary factors are not included in this. In addition, as you point out, there have been recent comprehensive reviews on the topic which would mean another review on these factors would be a repetition.<br><br>We have now clarified this in the introductory text on page 4. 'In addition to this, there is limited access in routine healthcare data to information on diet and these factors have already been explored in recent comprehensive reviews (8,9).' We have also added in the word 'clinical' to this sentence in the introduction on page 5 to clarify we are separating our statement from the recent reviews on factors such as diet. 'The last comprehensive summary review of meta-analytical studies examining clinical risk factors for pancreatic cancer was published nearly ten years ago and since then there have been a significant number of new systematic reviews looking at individual risk factors for pancreatic cancer (12)'
